# Supplementary material for: DnaJ mediates phage sensing by the bacterial NLR-related protein bNACHT25
Source: PLoS Biol. 2025 May 30;23(5):e3003203. doi: 10.1371/journal.pbio.3003203 (PMC12169576; doi:10.1371/journal.pbio.3003203)
Supplement: S1 Fig — Pairwise nucleotide identity (%) of ssRNA phages investigated in this study. (PDF) [file pbio.3003203.s001.pdf]

	Qβ	FrBurgundy	FrHibiscus	FrMerlot	FrSangria	MS2	FrBlood	FrHenna	FrSaffron
Qβ		94.3	94.4	94.1	94.2	37.5	36.9	36.9	37.1
FrBurgundy	94.3		99.9	97.0	94.7	37.4	36.9	36.9	37.1
FrHibiscus	94.4	99.9		97.0	94.8	37.3	36.8	36.8	37.0
FrMerlot	94.1	97.0	97.0		94.8	37.5	36.8	36.8	37.0
FrSangria	94.2	94.7	94.8	94.8		37.4	36.8	36.8	37.1
MS2	37.5	37.4	37.3	37.5	37.4		92.0	92.1	92.3
FrBlood	36.9	36.9	36.8	36.8	36.8	92.0		99.4	94.9
FrHenna	36.9	36.9	36.8	36.8	36.8	92.1	99.4		94.9
FrSaffron	37.1	37.1	37.0	37.0	37.1	92.3	94.9	94.9	

% Nucleotide identity
