# Supplementary figures and images for: DnaJ mediates phage sensing by the bacterial NLR-related protein bNACHT25
Source: PLoS Biol. 2025 May 30;23(5):e3003203. doi: 10.1371/journal.pbio.3003203 (PMC12169576; doi:10.1371/journal.pbio.3003203)

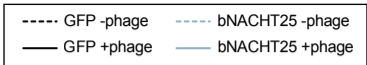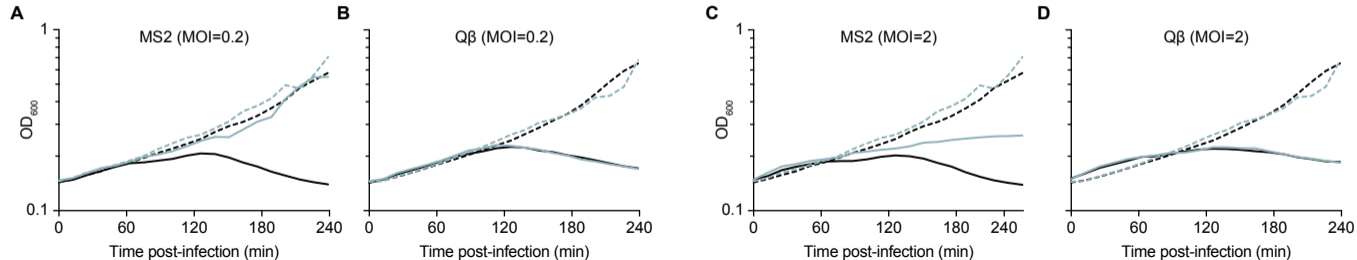

Supplement: S2 Fig — (A–D) Growth curves of E. coli expressing either GFP or bNACHT25. OD600 measurements began immediately following infection with the indicated phages at the indicated MOI. Data are representative of n = 3 biological replicates. The mean of n = 3 technical replicates for a representative experiment is shown. The data underlying this figure can be found in S1 Data. (PDF) [file pbio.3003203.s002.pdf]

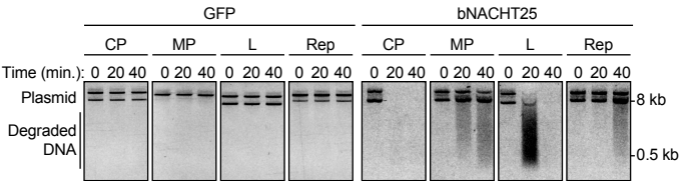

Supplement: S4 Fig — Visualization of plasmid integrity in E. coli expressing either GFP or bNACHT25 on the chromosome coexpressed with the MS2 protein indicated via an inducible plasmid. Plasmid DNA was harvested at indicated timepoints post-induction with IPTG. Data are representative images of n = 3 biological replicates. (PDF) [file pbio.3003203.s004.pdf]

**A**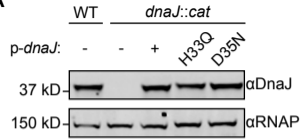**B**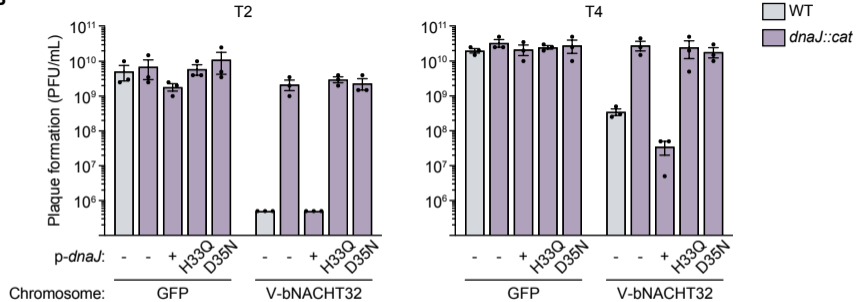**C**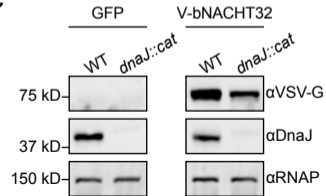

Supplement: S5 Fig — (A) Western blot analysis of cell lysates generated from E. coli with the indicated genotypes. p-dnaJ expresses dnaJ to WT levels without adding inducer. Data are representative images of n = 2 biological replicates. (B) Efficiency of plating of the indicated phage on WT or dnaJ::cat E. coli MG1655 expressing either GFP or VSV-G-bNACHT32 (V-bNACHT32) from the chromosome. Data plotted as in Fig 1B. Chloramphenicol acetyltransferase (cat). (C) Western blot analysis of cell lysates generated from E. coli with the indicated genotypes. Data are representative images of n = 3 biological replicates. The data underlying this figure can be found in S1 Data. (PDF) [file pbio.3003203.s005.pdf]

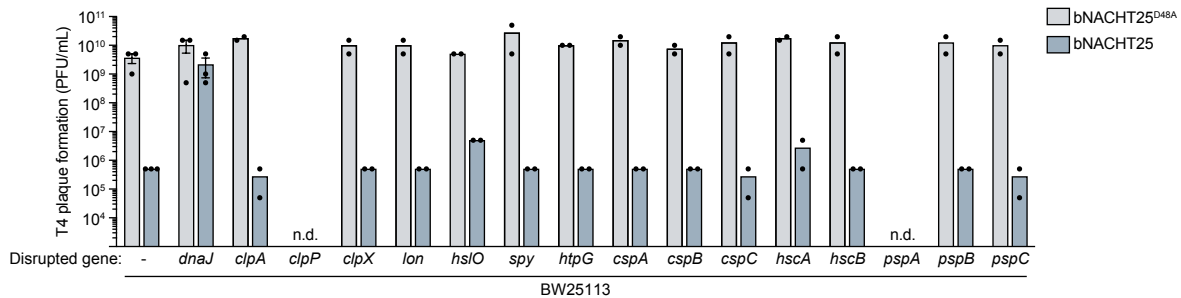

Supplement: S6 Fig — Efficiency of plating of phage T4 on E. coli BW25113 mutants containing plasmids expressing bNACHT25 or bNACHT25D48A. Not determined (n.d.) indicates the strain was unable to grow with one or both plasmids in the assayed conditions. Data plotted as in Fig 1B. The data underlying this figure can be found in S1 Data. (PDF) [file pbio.3003203.s006.pdf]

**A**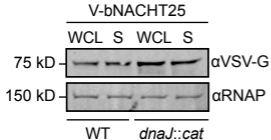**B**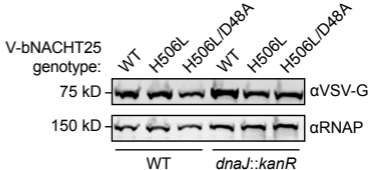

Supplement: S7 Fig — (A) Western blot analysis of Whole Cell (WCL) and Soluble (S) lysates obtained from E. coli WT or dnaJ::cat expressing VSV-G-bNACHT25 from the bacterial chromosome. (B) Western blot analysis of cell lysates generated from E. coli BW25113 expressing VSV-G-bNACHT25 with the indicated genotypes. All data are representative images of n = 3 biological replicates. (PDF) [file pbio.3003203.s007.pdf]

T4     $\lambda$ vir    MS2    Q $\beta$

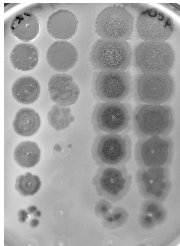

BW25113 + F'

T4     $\lambda$ vir    MS2    Q $\beta$

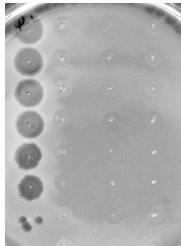

BW25113 + F'  
*dnaJ::kanR*

Supplement: S8 Fig — Efficiency of plating of phages T4, λvir, MS2, and Qβ on BW25113 WT or dnaJ::kanR. Data are representative images of n = 3 biological replicates. (PDF) [file pbio.3003203.s008.pdf]

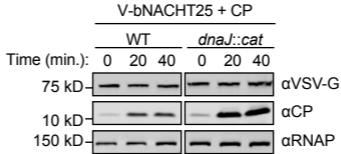

Supplement: S9 Fig — Western blot analysis of E. coli lysates from the indicated genotypes, timepoints, and conditions. Data are representative images of n = 3 biological replicates. (PDF) [file pbio.3003203.s009.pdf]

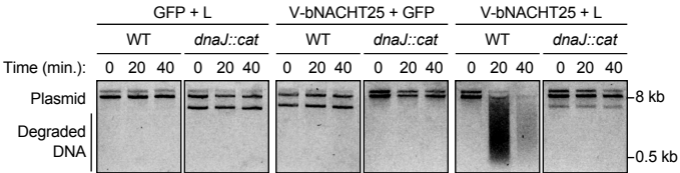

Supplement: S10 Fig — Visualization of plasmid integrity in E. coli with the indicated genotype at the indicated timepoints post-induction of L with IPTG. bNACHT25 is on the chromosome and L is on a plasmid. Data are representative images of n = 3 biological replicates. (PDF) [file pbio.3003203.s010.pdf]
