# Supplementary material for: DnaJ mediates phage sensing by the bacterial NLR-related protein bNACHT25
Source: PLoS Biol. 2025 May 30;23(5):e3003203. doi: 10.1371/journal.pbio.3003203 (PMC12169576; doi:10.1371/journal.pbio.3003203)
Supplement: S3 Fig — (A) Western blot analysis of bacterial lysates generated from E. coli expressing the indicated CP alleles and GFP from the chromosome. Bacteria were harvested 20-min post-induction with IPTG. (B) Western blot analysis of E. coli lysates generated from strains expressing bNACHT25 harvested at the indicated timepoints following infection with MS2 at MOI of 2 or induction of CP with IPTG. For (A–B), data are representative images of n = 3 biological replicates. (PDF) [file pbio.3003203.s003.pdf]

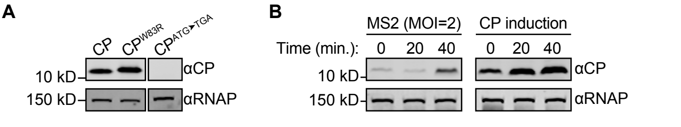

A
CP
CP W83R
CP ATG➤TGA
10 kD
150 kD
αCP
αRNAP
B
MS2 (MOI=2)
CP induction
Time (min.):
0
20
40
0
20
40
10 kD
150 kD
αCP
αRNAP
