# Supplementary material for: DnaJ mediates phage sensing by the bacterial NLR-related protein bNACHT25
Source: PLoS Biol. 2025 May 30;23(5):e3003203. doi: 10.1371/journal.pbio.3003203 (PMC12169576; doi:10.1371/journal.pbio.3003203)

Figure 1

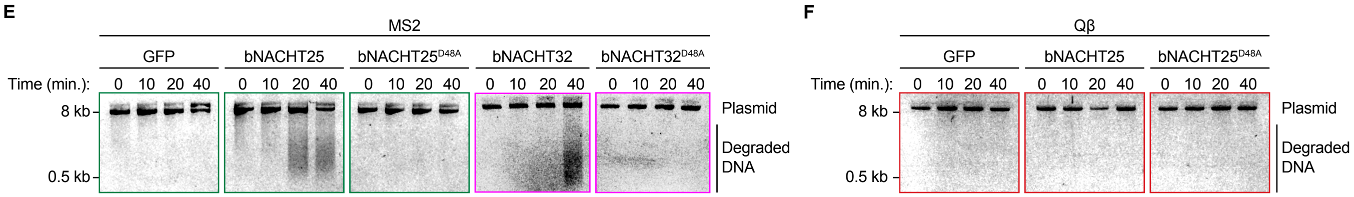

E
MS2
GFP
bNACHT25
bNACHT25D48A
bNACHT32
bNACHT32D48A
Time (min.):
0 10 20 40
8 kb
0.5 kb
Plasmid
Degraded DNA
F
Qβ
GFP
bNACHT25
bNACHT25D48A
Time (min.):
0 10 20 40
8 kb
0.5 kb
Plasmid
Degraded DNA

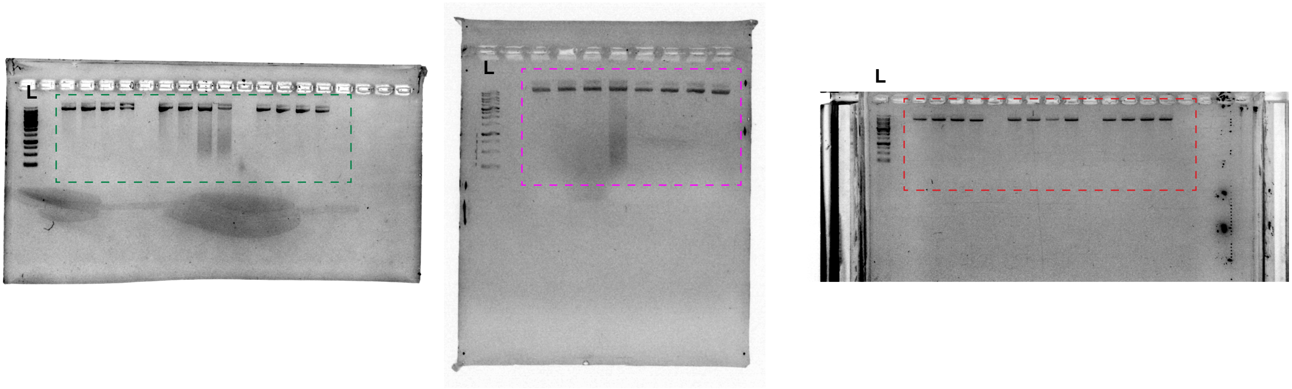

L
L
L

Figure 2

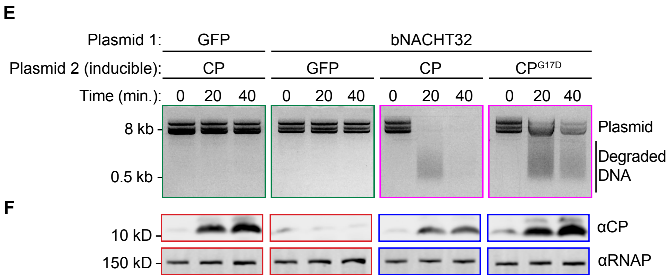
E
Plasmid 1: GFP bNACHT32
Plasmid 2 (inducible): CP GFP CP CP^{G17D}
Time (min.): 0 20 40 0 20 40 0 20 40 0 20 40
8 kb
0.5 kb
Plasmid
Degraded DNA
F
10 kD
150 kD
αCP
αRNAP

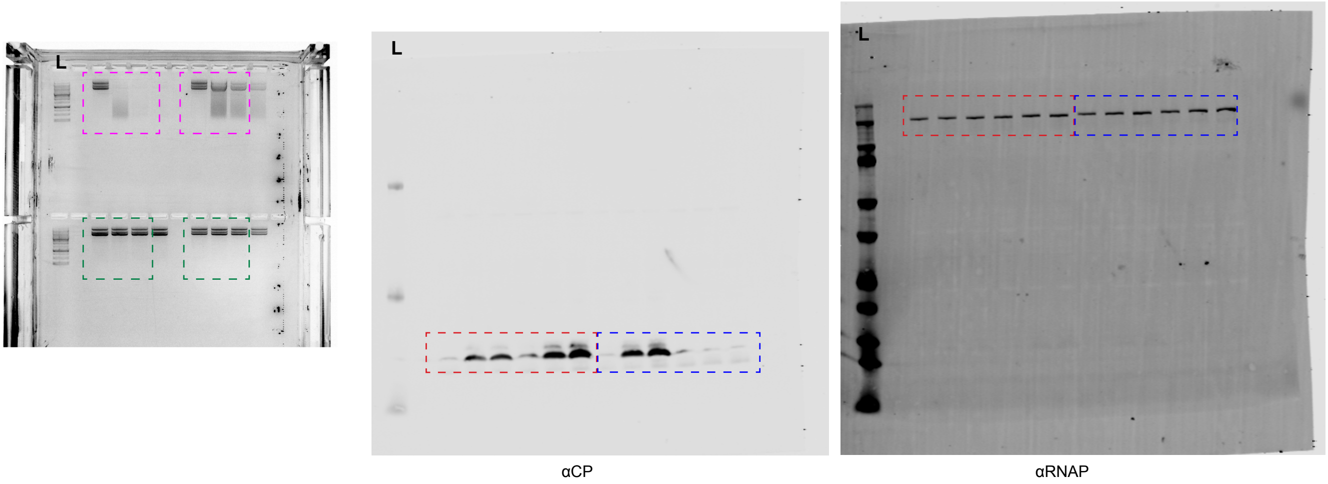
L
L
L
αCP
αRNAP

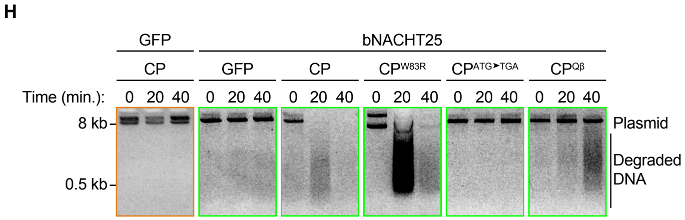
H
GFP bNACHT25
CP GFP CP CP^{W83R} CP$^{ATG➤TGA}$ CP^{Qβ}
Time (min.): 0 20 40 0 20 40 0 20 40 0 20 40 0 20 40 0 20 40
8 kb
0.5 kb
Plasmid
Degraded DNA

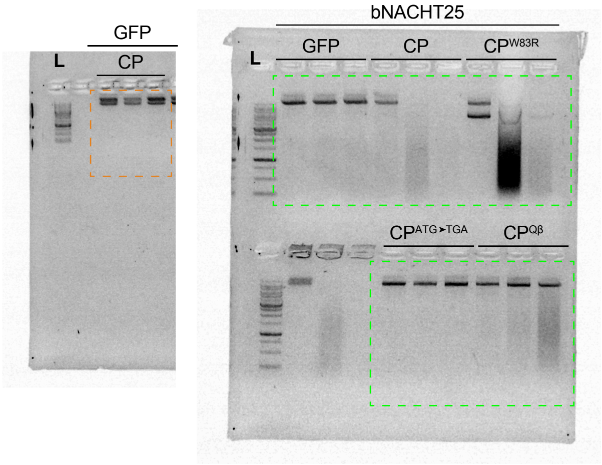
GFP
CP
L
bNACHT25
GFP CP CP^{W83R}
L
CP$^{ATG➤TGA}$ CP^{Qβ}

Figure 3

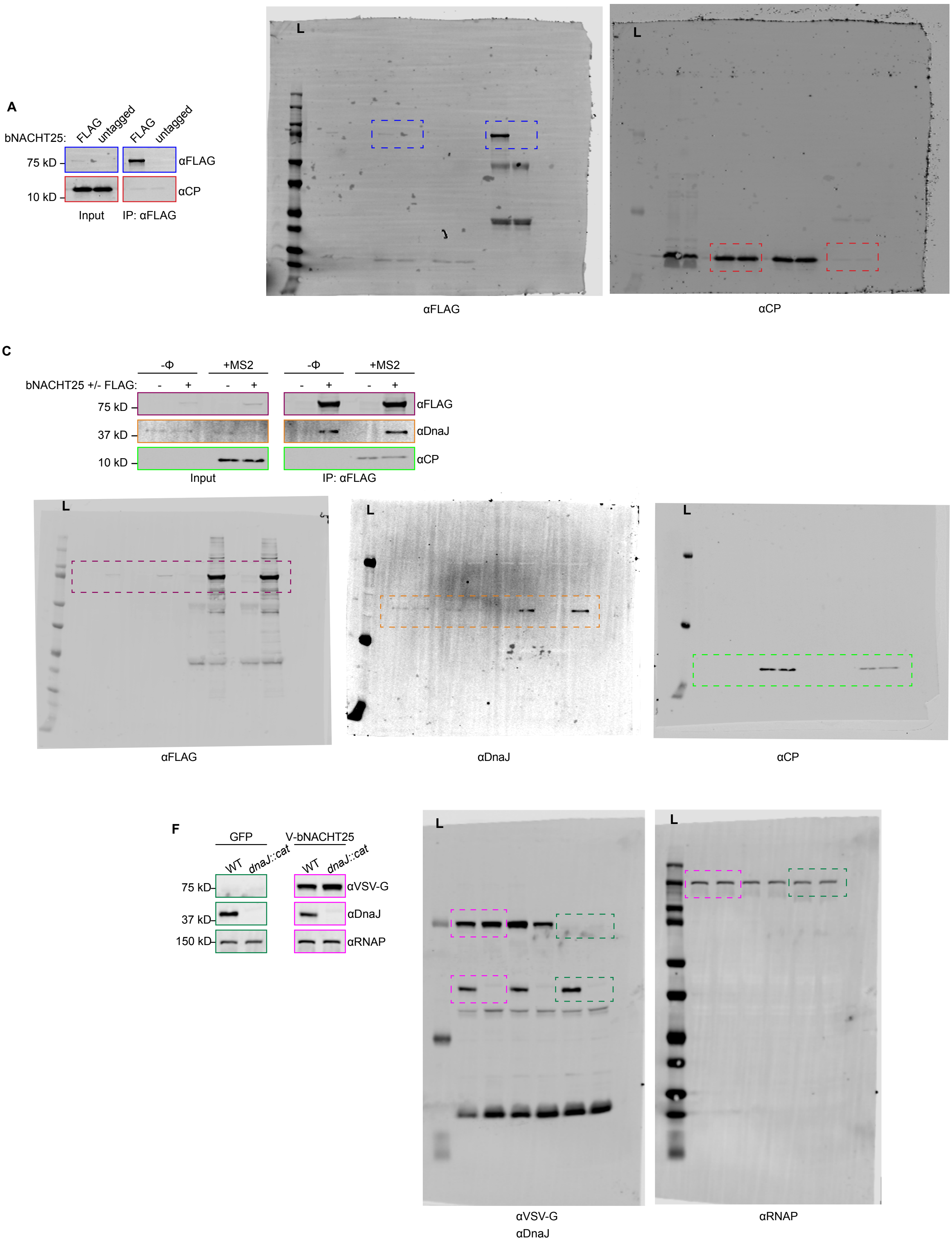

A
bNACHT25:
FLAG
untagged
FLAG
untagged
75 kD
10 kD
αFLAG
αCP
Input
IP: αFLAG
L
L
αFLAG
αCP
C
-Φ
+MS2
-Φ
+MS2
bNACHT25 +/- FLAG:
- + - + - + - +
75 kD
37 kD
10 kD
αFLAG
αDnaJ
αCP
Input
IP: αFLAG
L
L
L
αFLAG
αDnaJ
αCP
F
GFP
V-bNACHT25
WT
dnaJ::cat
WT
dnaJ::cat
75 kD
37 kD
150 kD
αVSV-G
αDnaJ
αRNAP
L
L
αVSV-G
αDnaJ
αRNAP

Figure 4

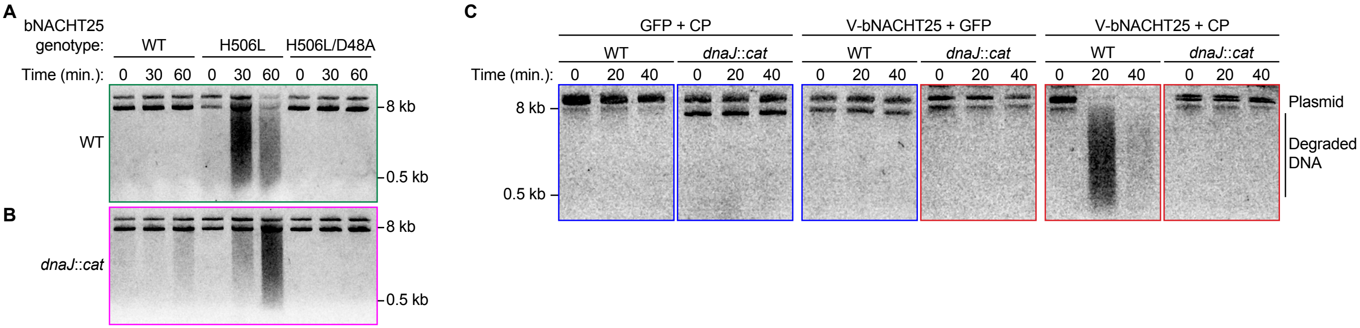
A
bNACHT25 genotype:
WT
H506L
H506L/D48A
Time (min.):
0 30 60 0 30 60 0 30 60
WT
8 kb
0.5 kb
B
dnaJ::cat
8 kb
0.5 kb
C
GFP + CP
V-bNACHT25 + GFP
V-bNACHT25 + CP
WT
dnaJ::cat
Time (min.):
0 20 40 0 20 40 0 20 40 0 20 40 0 20 40 0 20 40
8 kb
0.5 kb
Plasmid
Degraded DNA

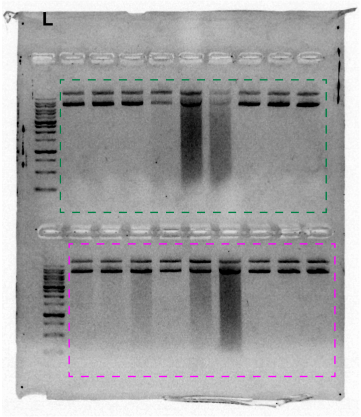
L

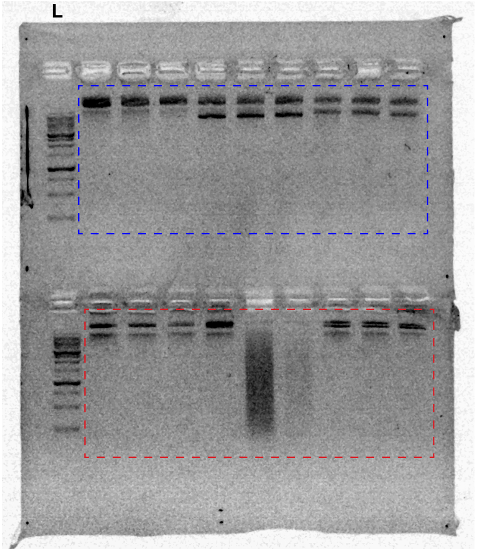
L

Supplementary Figure 3

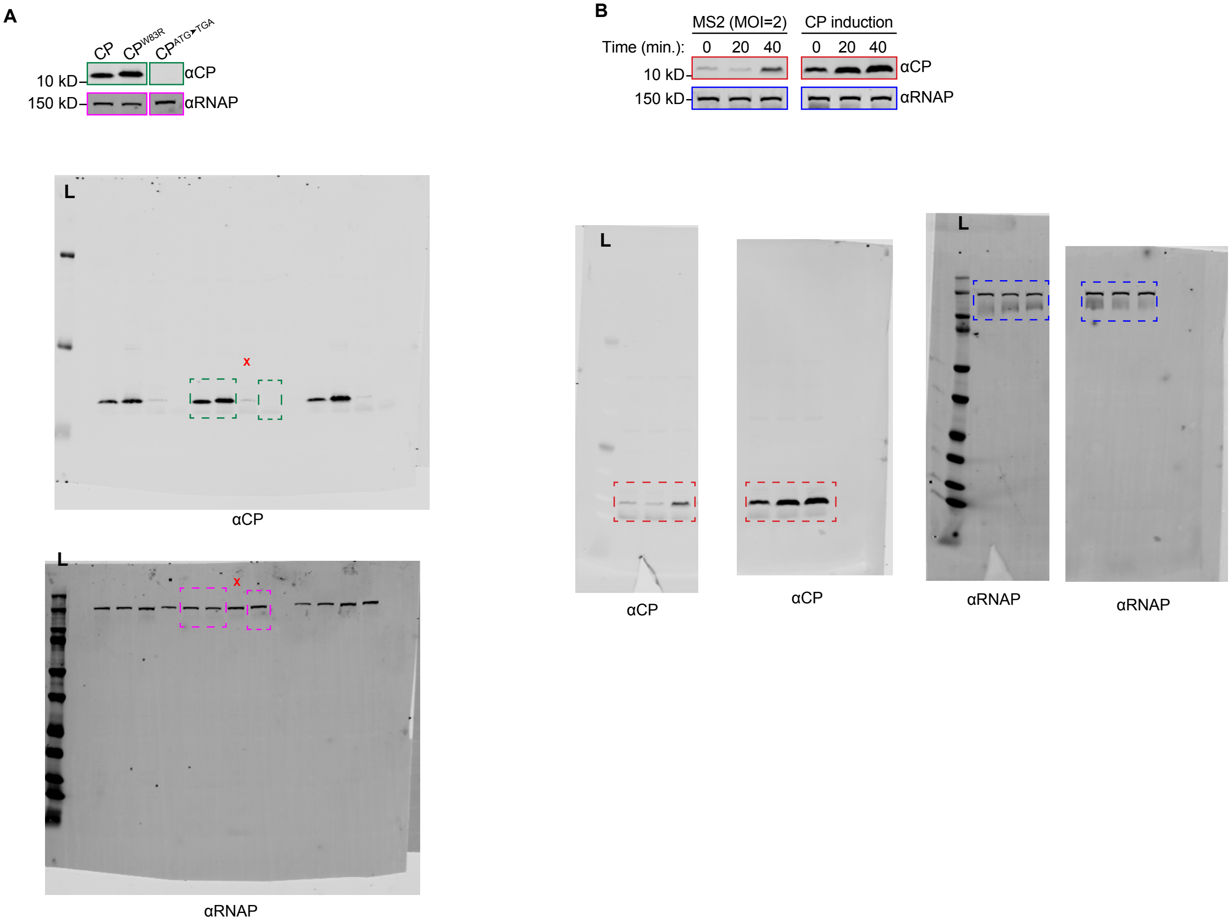

Supplementary Figure 4

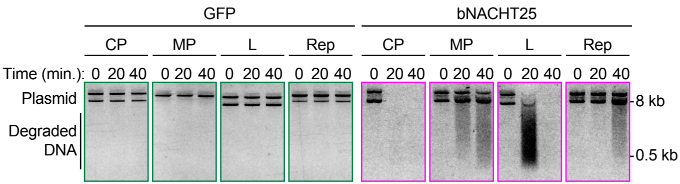

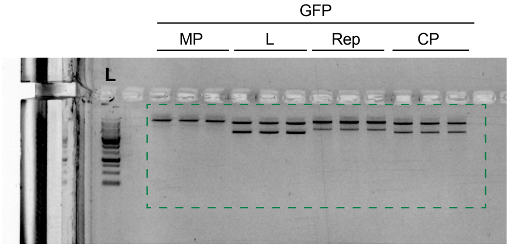

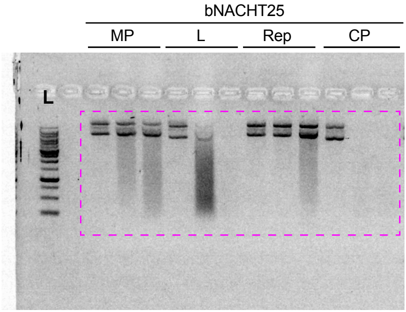

Supplementary Figure 5

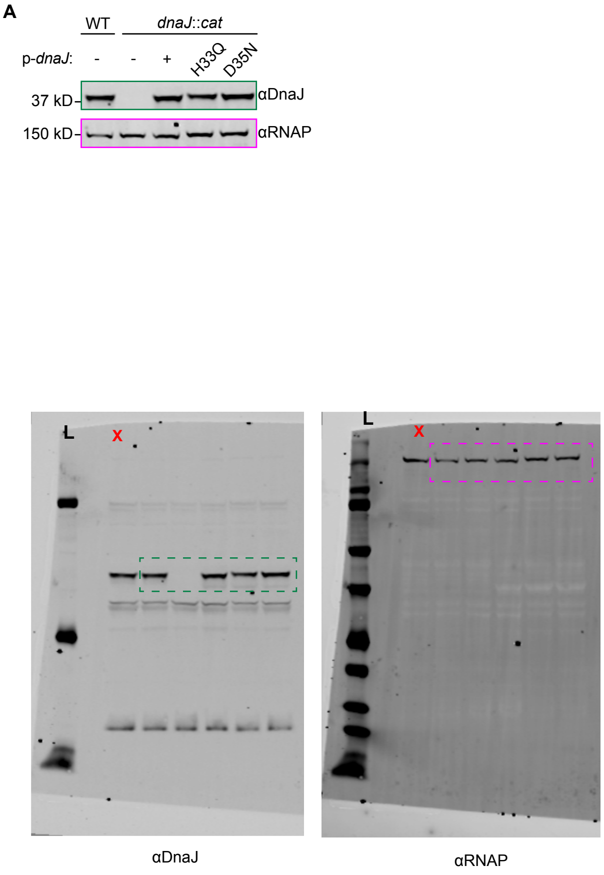

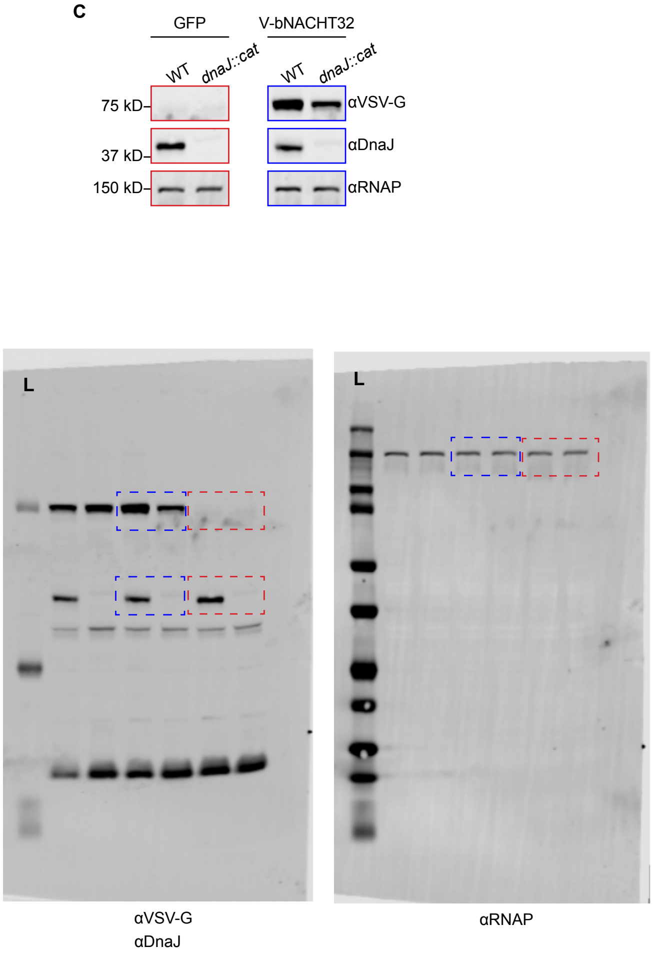

Supplementary Figure 7

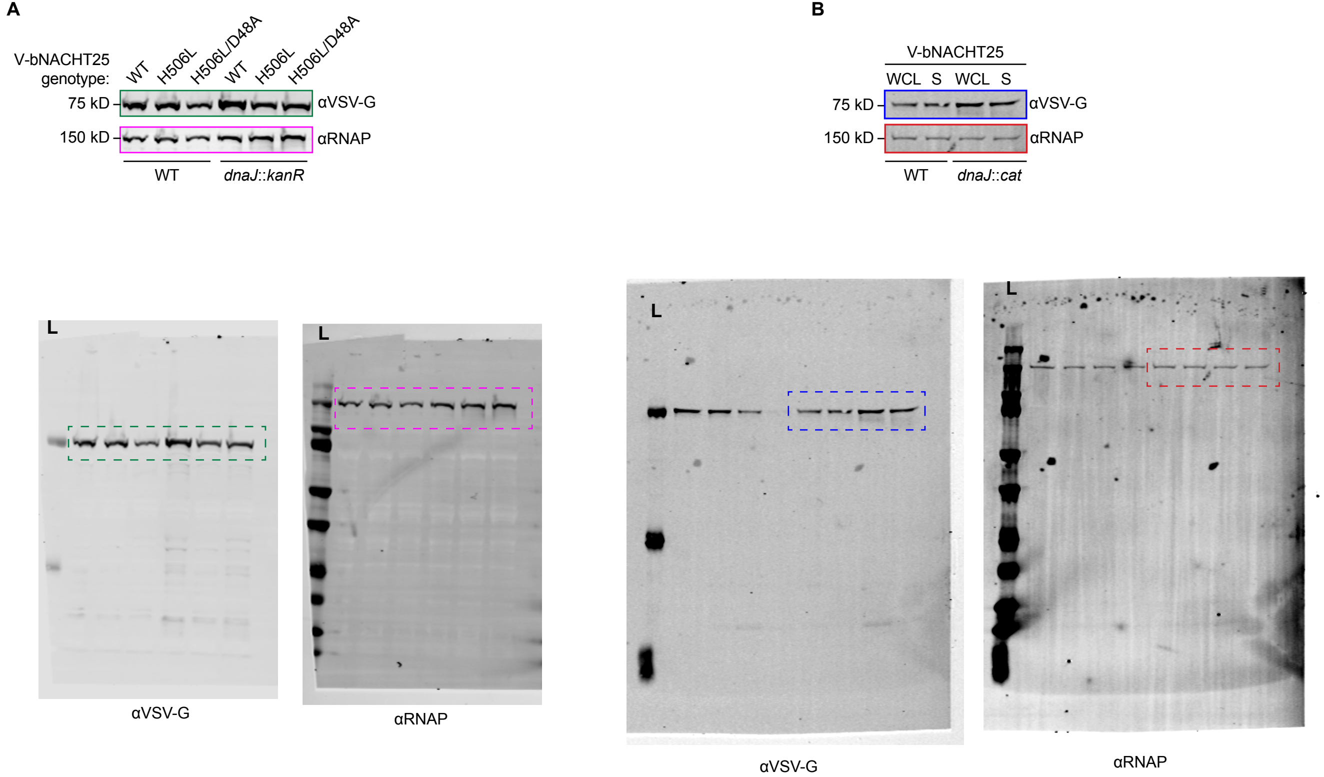

Supplementary Figure 9

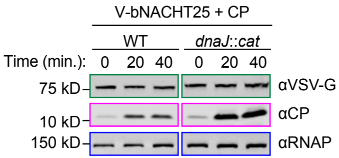

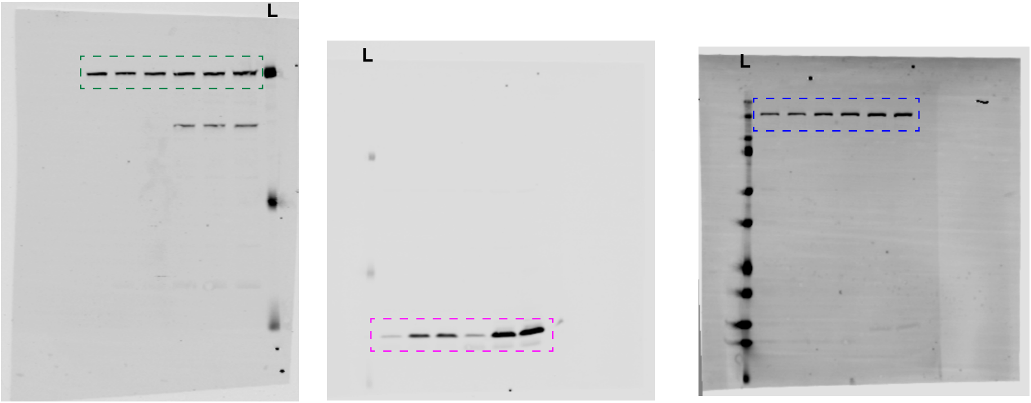

αVSV-G αCP αRNAP

Supplementary Figure 10

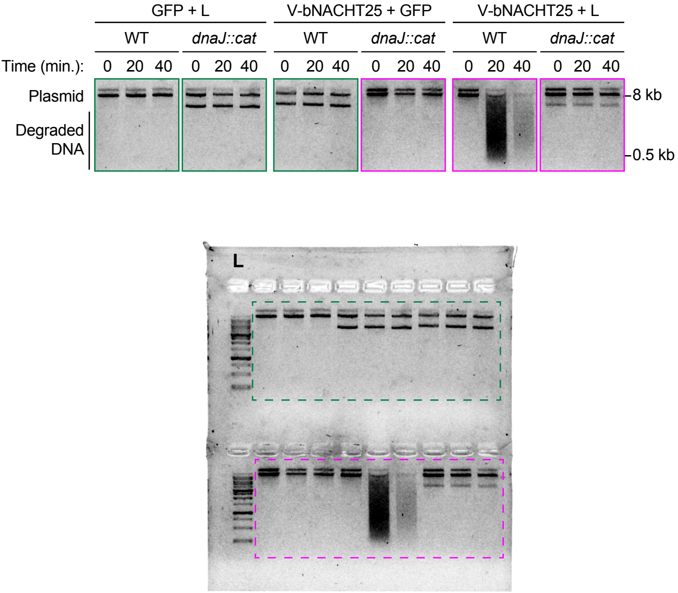

Supplement: S1 Raw Images — (PDF) [file pbio.3003203.s014.pdf]
