# Supplementary material for: DnaJ mediates phage sensing by the bacterial NLR-related protein bNACHT25
Source: PLoS Biol. 2025 May 30;23(5):e3003203. doi: 10.1371/journal.pbio.3003203 (PMC12169576; doi:10.1371/journal.pbio.3003203)
Supplement: Attachment — Submitted filename: Conte et al 2024- PLOSbio- Response to Reviewer.docx [file pbio.3003203.s016.docx]

**Response to Reviewers**

We thank the reviewers for their careful review of our manuscript and for their insightful comments and suggestions. Below, we address each comment and question in blue text. In addition, we have included revised copies of the manuscript with and without changes highlighted from the previous draft.

We appreciate and agree with the reviewers’ statements. The revised manuscript includes several additions that address major concerns of the reviewers and extend the findings of the manuscript. These are noted below in specific comment responses, including:

- We have now shown that hyperactive alleles of bNACHT25 that are stimulus independent are active in the absence of *dnaJ*, ruling out the hypothesis that *dnaJ* is simply required for bNACHT25 to fold/function.
- We have included additional controls, including complementation of the *dnaJ* knockout.
- We have performed bNACHT25 IPs during MS2 infection to show that CP does not IP with bNACHT25 while DnaJ does IP, supporting a physical interaction between bNACHT25 and DnaJ and providing a positive result for the experiment.
- We have included additional experiments that suggest a role for the DnaJ–DnaK complex in sensing phage, providing additional information on the mechanism of phage sensing through DnaJ.
- We have clarified our language on a few technical peculiarities of this story, including the switch from bNACHT32 to bNACHT25 and the lack of data for *dnaJ*-dependence of MS2 phage protection.

During the course of reviews, we also significantly improved other aspects of the manuscript, including changes to nomenclature, clarity of writing, and improved the figures.

**Reviewers’ Comments:**

**Reviewer #1 (remarks to the Author)**

In this paper, Conte et al. investigate how the anti-phage immunity pathway bNACHT is activated during phage infection. They find that MS2 phages with mutations in the coat protein (CP) are able to evade immunity. This result demonstrated that CP is necessary for triggering bNACHT, and the authors tested if CP is also sufficient by over-expressing the protein, in the absence of phage infection. They find that CP over-expression is toxic to the cells carrying the bNACHT system, triggering DNA degradation, a result which is consistent with bNACHT activation.

This is a very interesting result, but the next set of experiments did not clarify, and rather made more obscure, the mechanism of activation. This is because the authors could not find a direct interaction between CP and bNACHT proteins. When they performed MS to find proteins that interact with bNACHT, they find the chaperone DnaJ. But they did not perform validation experiments of the interaction, such as pull down experiments of using two-hybrid systems.

We have now provided additional evidence for the DnaJ–bNACHT interaction. Immunoprecipitation followed by western blot confirms that DnaJ interacts with bNACHT25 (**Fig 3E**).

And when they deleted dnaJ they did not find an effect on the phage MS2, which is where CP comes from, but on other phages such as T2 and T4.

Unfortunately, we failed to make clear in the initial submission that a peculiar detail of MS2 is that it cannot infect bacteria lacking the *dnaJ* gene. We have now clarified the text (**Lines 276-277**) and added an additional figure to demonstrate these data (**S7 Fig**). We do not know why MS2 cannot form plaques on bacteria lacking *dnaJ* and this has not been previously investigated in the literature.

This experiment also lacked a fundamental control, which is the complementation of the dnaJ mutant (which is interrupted by a chloramphenicol resistance cassette that could have polar effects on nearby genes), with a plasmid expressing the missing protein.

We have now fully complemented our *dnaJ* mutation with *dnaJ* expressed from an inducible promoter (**Fig 4D** and **S5B Fig**). These experiments also provided the opportunity to investigate *dnaJ* mutations that interrupt interaction with DnaK. Our newly included data show that DnaJ and DnaK work together to mediate bNACHT25 and bNACHT32 activation. These results help extend our understanding of the sensing mechanism for bNACHT25/32.

In the end, in the opinion of this reviewer, the paper starts with an interesting finding that is not followed up in depth, and little is learned about how bNACHT is actually activated. The data is quite thin for a PLoS Biology paper, but that is of course an editorial decision. The authors propose that DnaJ chaperone function on folding CP must generate the activation signal for bNACHT, but there is no data to support this, and DnaJ could be required for the folding of another protein that senses CP, for example.

We agree that the complete mechanism for bNACHT25/32 has yet to be fully elucidated, however, we feel that our manuscript is a meaningful advance in a challenging area. While there are many well-characterized examples of antiphage pathways that are activated by phage proteins, there are relatively few pathways that guard host processes whose mechanism is well understood. Further, our data explain how an antiphage pathway is able to sense both DNA and RNA phages.

We have clarified our language in **Lines 318-321** to emphasize that we do not know the direct substrate folded by DnaJ that is sensed by bNACHT.

Minor points:

-did the authors tried CP over-expression in liquid cultures? Is there toxicity as well?

We have now tested this and found that, yes, CP coexpression with bNACHT25 causes growth inhibition in liquid cultures in a *dnaJ*-dependent manner (**Fig 5C**).

-the rationale behind testing the CP G17D mutant is not clear.

We have now clarified this experiment in **Lines 150-152**. The finding that spontaneous MS2 mutants had the G17D mutation in CP led to the hypothesis that this mutation decreases bNACHT32 activation, allowing MS2 to avoid detection. Coexpression of CP^G17D^ with bNACHT32 activated bNACHT32 less than CP^WT^, supporting the hypothesis that CP activates bNACHT32 during infection.

-in Fig 4: is the difference for the dnaJ mutant (+/- bNACHT) not significant?

Indeed, the difference +/-bNACHT25 in the *dnaJ* mutant is not significant (p=0.39) in **Fig 4A**.

-Fig. 1 presents new data on bNACHT defense against additional Qubevirus, but it is hardly relevant for this manuscript. How different are the new Qubevirus at the nucleotide level?

The nucleotide identity of *Emesvirus* and *Qubevirus* phages is now presented in **S1 Fig**. These experiments show the breadth of RNA phage detection by different bNACHT proteins and that differences between these two clades of RNA phages are unlikely to be due to strain-level variation.

**Reviewer #2 (remarks to the Author):**

In Conte, et al, the authors follow up on the previous discovery that bNACHT25 degrades dsDNA and triggers cell death during infection by DNA and ssRNA phage. They demonstrate that Emesvirus (not Qubevirus) phages are targeted by bNACHT25 and other bNACHT systems. They show that MS2 phage escapers are mutated in the viral coat protein (CP) and that a CP mutant causes less cell death and dsDNA degradation than the wildtype coat protein. They demonstrate that expression of the coat protein from a plasmid is sufficient to activate bNACHT, but they don’t find a direct interaction between CP and bNACHT25. Using IP-MS, they find that DnaJ interacts with bNACHT and is required for phage targeting and dsDNA degradation by bNACHT. The authors state that bNACHT25 requires DnaJ to detect CP (rather than detect it directly) and propose that bNACHT monitors changes in the host that happen during phage infection. The author’s explanation is plausible, and the question they are addressing is interesting and important. However, the data they provide is not yet convincing enough to support their claims. Although DnaJ is clearly important for bNACHT activity, there is no evidence provided to show this is due to “sensing” as opposed to basic bNACHT functioning. Given that DnaJ is a chaperone protein, it may simply interact with bNACHT and enable its proper folding, as it does for many bacterial and phage proteins.

The authors show that bNACHT is still expressed in the absence of DnaJ, and that other chaperone mutations do not affect the phenotype. However, that does not demonstrate a role for DnaJ in detection, which is the most novel and important claim from this work.

In this reviewer’s opinion, more work needs to be done to convincingly show a role for DnaJ in detection. Given that some of the work has been shown before (i.e. bNACHT25 targeting MS2, mutations in structural proteins conferring phage escape, etc), additional mechanistic characterization of the bNACHT family could merit publication in PLoS, even if the sensing mechanism is not fully determined.

We agree with the reviewer that a formal alternative hypothesis that could have explained our data was that DnaJ is simply required for bNACHT folding and function independent of phage sensing. We have now ruled out this possibility by showing that a hyperactive, stimulus-independent allele of bNACHT25 is still able to degrade DNA in the absence of DnaJ (**Fig 5D and 5E**). The hyperactivating H506L mutation in the NACHT domain of bNACHT25 is predicted to destabilize the ADP-bound, inactive conformation of the protein. This allele is toxic to wild-type *E. coli* but can be expressed using an inducible promoter. Measuring bNACHT25-mediated nuclease activity for this mutant confirms that the effector function of bNACHT25 is not impacted by DnaJ. These data demonstrate that DnaJ is selectively required for upstream sensing of phage and CP.

**Reviewer #3 (remarks to the Author):**

The manuscript by Conte and colleagues investigates the mechanism of RNA phage detection by bNACHT proteins. They first show that selected bNACHT proteins could protect against MS2-like phages but not against related Qubevirus. Suppressor mutants of MS2 that evaded bNACHT01,12 or 32 antiphage activity were identified. A selected suppressor of bNACHT32 activity located in the coat protein (CP G17D) of MS2 was further studied and shown to be less efficient in activating bNACHT32 when coexpressed from a plasmid. The authors next showed that CP can also activate bNACHT25. In addition, they found that other MS2 proteins were also capable of inducing bNACHT25 activity, albeit less efficiently. Although no direct interaction between bNACHT25 and CP protein of MS2 could observed in vivo, IP followed by MS revealed several E. coli host proteins with an increased enrichment in the bNACHT25 IP samples, including the DnaJ co-chaperone. Further in vivo analysis revealed that the dnaJ gene was indeed required for bNACHT activation. Finally, the authors showed that bNACHT activation by MS2 L protein also required the dnaJ gene.

Although the involvement of the DnaJ cochaperone in the protection against phages is potentially interesting, this work is too preliminary and does not provide any mechanistic evidence for the role of DnaJ.
Major comments:
-It is not clear whether the nuclease activity of bNACHT25 or 32 is essential against MS2. Experiments monitoring PFUs in the presence of catalytic mutants are not provided.

We have now shown that phage protection by bNACHT25 and bNACHT32 is dependent on the catalytic activity of the nuclease domain (**Fig 1B**).

Accordingly, it should be tested whether the growth defect induced by bNACHT32 in the presence of CP or CP17D is dependent of its nuclease activity. These are important issues, which could significantly affect the later conclusion concerning the role of DnaJ.

We have now shown that the toxicity of CP with bNACHT32 is dependent on the nuclease activity of bNACHT32 (**Fig 2D**).

-Most, if not all of the suppressors identified have multiple mutations, not only in CP. How can the authors make sure that full suppression is only due to CP?

A significant challenge of working with RNA viruses such as MS2 is their elevated mutation rate. This problem is compounded by a lack of genetic tools for constructing targeted mutations. Indeed, our escaper phages contained multiple mutations, however, we consistently found the same CP^G17D^ mutation in 3 different MS2 escapers from 2 different parent lineages. Further, the additional mutation in ESC2.1 is silent for the Mat protein. These data provide a strong argument for the mechanism explaining MS2 escaper phages, but are subject to technical limitations.

-The mechanism by which the CP G17D suppressor substitution bypasses bNACHT32-mediated defense has not been demonstrated. The switch from bNACHT35 to bNACHT25 halfway is not obvious when considering the lack of suppressors identified on bNACHT25.

We agree that the exact consequence of the G17D mutation has not been elucidated, however, the consequence of this mutation on bNACHT32 activation has been clearly shown (**Fig 2D and 2E**). We hypothesize that we cannot generate escaper phages from bNACHT25-expressing bacteria because this system has more robust effector activation than bNACHT32, as described in the manuscript text (**Line 172-174**). During the course of our revision, we tried again to generate escaper phages on bNACHT25-expressing bacteria and were unsuccessful.

-Since the aim of this work is to determine the mechanism of RNA phages detection by bNACHT, it is not clear why T4 or T2 and not MS2, were used to assess mutations in enriched candidates obtained by MS. How does MS2 react on these mutants?

We now provide data showing MS2 is unable to replicate in bacteria lacking *dnaJ* (**S7 Fig**). This is both a technical limitation of the study and a potentially important finding. We have clarified these findings in the text (**Lines 276-278**). A beneficial consequence of our switching to T2 and T4 phages is demonstrating just how broadly relevant our findings are outside of RNA phages.

DnaJ might be involved in multiple stages of the MS2 lifecycle. Previous publications have shown that DnaJ plays a role in lysis by binding L protein of MS2 and there could be other aspects of virion production that require DnaJ. Alternatively, DnaJ may has been implicated in replication of certain plasmids, and perhaps problems with F replication or pilus production underlie our data. In either circumstance, these data clearly show an intimate interaction between DnaJ and MS2 phage replication that may explain why DnaJ is a perfect host component for bNACHT25 to monitor.

-Several of the MS enriched candidates identified in this work are chaperone proteins (i.e., IbpA/B, DnaK or DnaJ) typically pulled down with aggregation sensitive proteins. Is overexpressed bNACHT25 fully soluble in the absence of dnaJ vs wild-type?

We now provide a crucial experiment to demonstrate that the bNACHT25 protein is fully functional in *dnaJ*-deficient cells (**Fig 5D and 5E**). We have used an inducible system to express a hyperactive allele of bNACHT25. In these experiments, bNACHT25 is active in a stimulus-independent manner and disruption of *dnaJ* did not impact nuclease activity (**Fig 5D and 5E**). These data complement data already found in the manuscript showing that bNACHT25 is similarly expressed in wild-type and *dnaJ*-mutant strains (**Fig 4C**).

-There is no solid experimental evidence in vivo and none in vitro, demonstrating the mechanism by which DnaJ contributes to the protection against RNA phages. Since DnaJ is essential for MS2 lysis, it is possible that bNACHT merely titrates out the available DnaJ, thus preventing MS2 growth. In addition, it remains to be demonstrated whether the observed DnaJ effect is linked, or not, to its DnaK-cochaperone activity.

We have now addressed this point by introducing a plasmid expressing *dnaJ* to *E. coli* *dnaJ*::*cat*. While DnaJ^WT^ complements the phage defense phenotype of bNACHT25 and bNACHT32 in *dnaJ*::*cat* bacteria, introduction of the H33Q and D35N mutations to the J-domain of DnaJ abrogates the complementation. These mutations have previously been shown to prevent DnaJ from interacting with its cochaperone DnaK. These data suggest that the cochaperone activity of DnaJ is important for its role in mediating bNACHT25 and bNACHT32 activation and are detailed in **Lines 247-253** and **Fig 4D** and **S5B Fig**. We have also detailed the experimental limitations that precluded analysis of MS2 phage infection of *dnaJ* deficient bacteria (**Lines 276-278**).

Importantly, one of the main functions of the DnaJ/DnaK machine is to control the heat shock response (HSR) that regulates hundreds of genes in E. coli. Therefore, it remains to be determined whether the involvement of DnaJ is direct or indirect, via the HSR.

We agree that the molecular details underpinning how DnaJ/K specifically interact with bNACHT25 and are impacted by phage remain to be completely elucidated. However, our manuscript provides an important advance to understanding by identifying and characterizing the key players in what is clearly a complicated molecular mechanism.

Minor comments:
-There is no information provided about the chromosomal VSV-G-bNACHT25 construct used in this work.

The description of the *lacZ::VSV-G-bNACHT25-kanR* construct is now expanded and improved in **Lines 242-243**. The construction is also described in the Materials and Methods (**Lines 623-627**) and the DNA sequence inserted at the *lacZ* locus appear in **S3 Table** (strain AW3227).

- It is not clear why IP were not performed during phage infection.

In response to this critique, we have performed the suggested experiment and these data are consistent with our previous findings. CP did not immunoprecipitate with bNACHT25 during MS2 infection (**Fig 3E**). Additionally, this assay adds more support for DnaJ interacting with bNACHT25 in the presence and absence of phage infection.

**Reviewer #4 (remarks to the Author):**

This is a very interesting manuscript with the potentially exciting finding that DnaJ plays a role in the function of an anti-phage defence protein. This result could be publishable in PloS Biology, but the current manuscript has a number of deficiencies that must be addressed. In general, a deeper analysis of the data is required, and additional experiments need to be performed in order to clarify the mechanism of these systems and the role of DnaJ.

In Fig. 1b and c, the quantitation in part c does not seem to be consistent with the zones of clearing seen in b. For example, parent 2 and escaper 2.1 look the same in b, but are reported to have a greater than 10-fold difference in c.

We apologize for the confusion with the quantification of our MS2 escapers. In our images of phage spot plating, the first dilution is normalized based on the titer of the phage in order to highlight the difference +/- the defense system. For the parent phages, the most concentrated spot in the dilution series is a 10^-1^ dilution. For the escapers, the most concentrated spot in the series is undiluted lysate. Our PFU/mL calculations take these dilutions into account, which is why the PFU/mL values for the parents are 10-fold higher than the escapers, despite appearing to be similar titers in the plate images. Plate amplification protocols or phage genotypes sometimes lead to differences in phage titers. We have not investigated why these escapers have a lower titer but it may be due to a fitness cost of the escaper mutations.

We have fixed this issue in two ways: 1. We have now reported phage protection in terms of fold defense, which normalizes for the titer of the phage in the absence of the system (**Fig 2C**), and 2. We have noted the differences in the first spot in each dilution series in the **Fig 2B** legend.

In the materials and methods, the authors describe their quantification procedure. It sounds odd to me. Why not simply plate an appropriate dilution on a whole plate and count the plaques. In this way, one can always be counting at least 100 plaques to get statistically significant data. A problem with this approach is that in the absence of defence, plaques may be large and in the presence of defence, plaques may be very small. Thus, a simple plaque count can be misleading as you are counting large plaques and small plaques as the same thing (i.e. one plaque). If this is the case, spotting can provide a more realistic qualitative view because small plaques will yield a smaller overall zone of clearing. It is complicated, though, to quantitate zones of clearing. Performing phage growth assays in liquid can be more effective in some cases. This involves growing phages with and without the defence system in liquid culture for some period of time and then titering the phages produced during the growth period on a strain without a defence system. The authors should think about this issue as the quantitation of their plaquing data has important ramifications for interpretation of the experiments.

We have clarified our methods for quantifying in PFU. We agree that for some defense systems, it is appropriate to consider reduction in plaque **size** as opposed to plaque **number**. However, in our case, we see a dramatic decrease in plaque **number** when bNACHT systems are present. Counting PFU using 10-fold dilutions of lysate is a well-accepted method in the field and produces high-quality data. A possible source of confusion here could be the details of our methods for when zero defined plaques can be visualized on the highest concentration of phage. In this case, it is necessary to estimate the limit of detection for the assay. While plating a full plate would indeed decrease the limit of detection, this is not necessary for any of our comparisons.

In the abstract, the authors state, "Coat Protein (CP) was sufficient for activation." I don't think that the authors have proven that CP is sufficient for activation during a phage infection. They have shown that co-expression of NACHT proteins and CP causes DNA degradation and reduced cell viability. However, DnaJ is required for this and there appears to be no direct interaction between CP and NACHT25. There is clearly more going on. The authors show that phages bearing the G17D substitution in CP replicate ~10X better than WT in the presence of NACHT32 (Fig. 1c). However, these phages also replicate at least 10X worse in the absence of NACHT32.

We have now clarified the abstract to distinguish between the phage escaper mutants and the genetic assay. We thank the reviewer for pointing out this point of confusion. Indeed, the escaper mutants did not show that CP was sufficient, instead, the genetic assay showed that CP was sufficient.

The effect of this mutation could be an indirect result caused simply by less robust phage replication. Given that the authors identified 6 other substitutions in CP that lead to escape from NACHT systems, it would be informative to report the behavior of these mutants. Did they also attenuate replication in the absence of a NACHT system? Do they affect replication on NACHT32? Do all these substitutions map on a single surface of CP? If they do, this could suggest an interaction interface with another protein (DnaJ?). In line 160, the authors state that the "G17D mutations in CP allow MS2 to evade detection". This is not true. The phages partially evade detection. Fig. 1c indicates that NACHT32 causes a 1000-fold drop in phage titer. The G17D substitution only increases titer by 10-fold. The authors must generally avoid wording suggesting that CP is the whole story, and that CP substitutions overcome the defence system. This point is underscored by the fact that L protein and MP also induce DNA degradation by NACHT25.

We have now edited our language for accuracy.

The bNACHT32 escapers that had mutations in CP all had the G17D mutation. These escapers are specific for bNACHT32. The CP mutations found in the bNACHT02 and bNACHT12 escapers, such as K44R and V68A, are in a different location on the protein (left) and will be the subject of future analysis.

*Crystal structure of MS2 CP in complex with cognate RNA (1ZDJ). Residues mutated in MS2 escapers of bNACHT32 (G17) and bNACHT02/12 (K44 and V68) are labelled.*

I do not understand the switch to NACHT25 for plasmid based DNA degradation assays. The authors say that "NACHT25 provided provided more robust effector activation in response to phage (Fig. 1d)". However, Fig. 1d does not show data for NACHT32. Thus, the reader does not know if NACHT32 activates DNA degradation at all.

This concern is now addressed in **Fig 1E**. While bNACHT25 degrades DNA by the 20-minute timepoint post-infection, bNACHT32 seems to only become activated 40-minutes following infection by MS2. We have further addressed this confusion by altering the language at **Lines 118-119**.

The vast difference between the level of DNA degradation activated by phage infection and that activated by plasmid co-expression is a big red flag to me. This supports the hypothesis that the plasmid assay is not reflecting what is really happening during phage infection. The authors also do not show whether the G17D substitution changes the replication of MS2 on NACHT25. If it does not, is NACHT25 really a valid model for studying NACHT32?

This is an interesting point, and one that we have thought deeply about. We know that bNACHT25 and 32 protect against the same panel of phages, share 81% identity at the nucleotide level, and share 97% similarity at the protein level. Thus, we treat bNACHT25 and bNACHT32 as homologs. The only difference we have found is that bNACHT32 seems to become activated later in infection, and this may contribute to our lack of success isolating bNACHT25 escaper phages, despite exhaustive screening and selection. It is possible that mutations that allow for bNACHT25 escape are so destabilizing to MS2 that they are unable to form plaques. Despite this limitation, we were able to use the robust activity of bNACHT25 to our advantage in the plasmid degradation assays. These assays provide a reduced complexity system and higher gene dosage than phage infection, allowing us to assess the effects of phage proteins on bNACHT25 activation.

In line 168, the authors state, "Expression of CPW83R, which is unable to oligomerize beyond dimerization and limits capsid assembly25, similarly induced DNA degradation (Fig 3a)". This mutant induces degradation much more slowly than wild-type. This difference may be important and should not be ignored. The authors must also test the degradation of the G17D mutant by NACHT25. It may be degraded at the same level as the W83R mutant. This would completely change the interpretation of this experiment. The authors must think more about these results and discuss the implications.

We thank the reviewer for pointing this out and agree that the W83R does decrease DNA degradation (but not cell death) caused by CP activation of bNACHT25. We have now discussed the potential implications of this observation on our model (**Lines 198-199**).

The finding that DnaJ influences the activity of the NACHT systems is very interesting. However, the authors have failed to directly connect the lack of DnaJ to defence against phage MS2 infection. The infection and escaper mutation experiments are done with NACHT32, but the DNA degradation assays in dnaJ∆ mutants are done with NACHT25. As alluded to above, these should be done with NACHT32 also. More importantly, the effect of DnaJ on MS2 replication in the presence of NACHT defences systems should be measured. The authors say that MS2 could not form plaques on the DnaJ mutant, but why? If it is due to inhibition of lysis (seems likely), then infections could be performed in liquid. Cells could be spun down at various timepoints and lysed by some other means to release intracellular phage particles. The authors could also assay DNA degradation during infection. Knowing which step in the phage lifecycle is blocked in the absence of DnaJ could provide some clues about the activation mechanism of the NACHT proteins.

The inability of MS2 to infect a *dnaJ* knockout is an unfortunate limitation of this study that we do not fully understand. DnaJ involvement in MS2-induced lysis may be playing a role, and we have now included a figure depicting phage infection in WT vs *dnaJ*::*kan^R^* backgrounds (**S7 Fig).** A strength of this study is that MS2 protects against both DNA and RNA phages, and the finding that *dnaJ* is required for protection against T2 and T4 may imply that the ability to monitor DnaJ enables bNACHT25 to sense diverse phages. We investigated the role of DnaJ in MS2 detection using coexpression assays. We found that activation of bNACHT25 by the MS2 proteins CP and L is completely dependent on DnaJ (**Fig 5A-5C** and **S9 Fig**), suggesting that DnaJ may be required for bNACHT25 detection of MS2.

Although the findings in this paper, especially the potential role for DnaJ, are tantalizing and intriguing, the study is ultimately unsatisfying because the proposed mechanism is so vague. The authors should focus on the MS2 phage life cycle more, and rely less on the non-physiological plasmid-based DNA degradation assay. The simplest explanation for the role of DnaJ is that phage infection leads to an increase in DnaJ concentration, then this excess DnaJ binds and activates NACHT.

This idea is not totally crazy as it has been shown that lambda infection increases the levels of heat shock proteins (PMID: 6460750, PMID: 6460752). The authors should monitor DnaJ expression during MS2 infection and look at the effect of DnaJ on in vitro NACHT activity. The CP mutants may manifest their effects by causing a smaller increase in DnaJ expression. The observation that these mutants do not replicate as well as wild-type is consistent with the idea that they don't induce DnaJ as much.

This is an interesting hypothesis. We have tested this by measuring DnaJ protein expression during MS2 infection. We did not observe an increase in DnaJ concentration by western blot following infection by MS2 (**Fig 3E**).

The proposed experiment looking at the effect of DnaJ on *in vitro* bNACHT25 activity is one that we have considered, but the suggested *in vitro* work is outside the scope of the present study. The proposed experiment would likely require an indeterminant amount of time due to potential involvement of cochaperone DnaK, its nucleotide exchange factor GrpE, and because we have not successfully produced recombinant bNACHT25.

Other Issues:

I believe that "suppressor" is the wrong term to use for the mutant phages isolated that are not blocked by defence systems. These phages are escaper mutants as they escape from the effects of the defence systems. "Suppressor" usually refers to a mutation that suppresses the effect of another mutation in the same organism. I strongly recommend that the authors use "escaper" mutant instead of "suppressor".

We thank the reviewer for their suggestion and have changed our language for clarity throughout the manuscript (and the response to the reviewers!).

Line 182: remove "contractile". Lambda is not contractile.

Thank you for pointing this out, this has been corrected in **Line 190**.

Line 188: "Of note, the inducible vector in the genetic assay used expressed CP slightly higher than MS2 infection (Extended Data Fig 2c)." I do not think that "slightly" is the right word here. As I see it, there is a large difference in expression, especially at the early timepoints. Again, these data highlight important differences between the plasmid expression system and the conditions of phage infection. Ignoring these differences will prevent a true understanding of what is really happening during phage infection.

We have removed the term slightly.

Did the authors test for an interaction between CP and NACHT32? They have presented no evidence that CP activates NACHT25 in the context of a phage infection. Do the phages that escape NACHT32 also escape NACHT25? This is a crucial question. In addition, the IP assay has no positive control. What if the interaction between CP and NACHT25 is too weak to be detected under the conditions used. Since there is no positive result for comparison in these assays, they are essentially meaningless. To rule out an interaction between 2 proteins, one has to be more rigorous. The authors must use more than one assay, and each must include meaningful positive controls.

We agree that this assay requires a positive result to compare to. We have now performed an IP during phage infection to support the claim that CP does not form a complex with bNACHT25 (**Fig 3E**). We also blotted for DnaJ, and have shown that DnaJ interacts with bNACHT25, providing a positive result for the experiment (**Fig 3E**).

For the IP-MS experiments, it is not clear why some proteins were chosen for further analysis and others were left out. Looking at Supp Table 2, it seems that there are lots of other enriched candidates. The authors need to explain this.

We have now provided a more thorough explanation in **Line 229-230**. We chose proteins for further genetic analyses based on the following factors: 1. For experimental simplicity, we omitted proteins encoded by essential genes and genes that were absent from the Keio Collection of *E. coli* gene deletions, and 2. We chose to focus on proteins that were enriched in both of our two IP-MS experiments.

In the DNA degradation assays, why do the undegraded samples have 2 bands in some lanes and one band in other lanes.

We are uncertain, however, we believe this is due to different plasmid topologies, such as differences in supercoiling states that are purified by the plasmid miniprep protocol.
