# Supplementary material for: DnaJ mediates phage sensing by the bacterial NLR-related protein bNACHT25
Source: PLoS Biol. 2025 May 30;23(5):e3003203. doi: 10.1371/journal.pbio.3003203 (PMC12169576; doi:10.1371/journal.pbio.3003203)
Supplement: Attachment — Submitted filename: Conte et al - 3rd sub - PLOS Biology_Response to Reviewers.docx [file pbio.3003203.s018.docx]

**Response to Reviewers**

We thank the reviewers for their comments and suggestions. Below, we address each critique in blue text. In addition, we have included revised copies of the manuscript with and without changes highlighted from the previous draft.

**Reviewers’ Comments:**

**Reviewer #2:**

In this revision, Conte, et al have added important and clever experiments that further support a role for DnaJ in bNACHT detection. Among other experiment, they use a hyperactive NACHT25 mutant that requires no stimulus and show that bNACHT is still active in the absence of DnaJ, indicating a role for DnaJ in sensing rather than bNACHT folding. This reviewer believes that the new data makes the work compelling and rigorous enough to be published in PLoS Biology. However, they should revise their language throughout the article to reflect the fact that the role of DnaJ in sensing (rather than degradation) is only clear when they show Figure 5.

We agree and have adjusted our language prior to explaining Fig. 5A to indicate that DnaJ is necessary for “protecting against phage” instead of “sensing phage” (Lines 242, 265, and 271). We have also changed the order of the text and figures to introduce the hyperactive bNACHT25 experiment earlier.

Likewise, it still remains unclear if DnaJ has a direct or indirect role in sensing. Given its basic function, and how little is still known about the sensing mechanism, it is still possible that DnaJ is simply involved in folding other factors (phage proteins or another sensing component) that enable detection. Their proposed model that bNACHT detects CP proteins through its interaction with DnaJ is exciting and compelling, but they should acknowledge the possibility that DnaJ might play an indirect role in the results and discussion.

We acknowledge that the full sensing mechanism has yet to be discovered, and for this reason, have changed out submission to a “Discovery Report.” This is a format designed for interesting studies that may not have a complete molecular mechanism. We have also now included a paragraph within the discussion to explain the limitations of our study to acknowledge alternative hypotheses and outstanding questions like those described (Lines 384-393). Finally, while our IPs suggest that bNACHT25 and DnaJ associate in the same complex, it is true that their interaction may not be direct, but rather may be bridged by another molecule. We have adjusted our model (Fig. 5F) to represent this uncertainty.

**Reviewer #3:**

The role of DnaJ and it chaperone/cochaperone partners is phage detection is very interesting.

The authors have made significant changes and added several experiments to further address the role of DnaJ in vivo. However, the mechanism by which DnaJ mediates bNACHT activation by phage proteins is still not provided. As it is, we are left with an interesting in vivo observation but no real mechanistic breakthrough.

-There is no experiment supporting the proposed role of DnaJ in vitro.

We have attempted to construct an *in vitro* assay to measure bNACHT25 activation using recombinant bNACHT25 to rule out DnaJ-dependent protein differences. We performed an experiment in which we incubate purified bNACHT25 or bNACHT25^D48A^ with purified plasmid DNA and T4-infected cell lysate from WT and *dnaJ-*mutant *E. coli.* We hypothesized that the stimulus that bNACHT25 receives during phage infection would be present in the infected lysate and would be able to activate bNACHT25 *in vitro*. While we were able to purify bNACHT25, we did not observe any convincing bNACHT25-dependent DNA degradation in T4-infected wild-type *E. coli* in this assay (left).

We think our results reflects a technical challenge. It could be that we have not identified the appropriate buffer conditions or that even when ATP is supplemented into this reaction that the cell lysate quickly hydrolyses the NTP to NDP/NMP. Given the time constraints of preparing this revision and that further optimization would require an indeterminant amount of time, we have elected to not further refine these experiments. We have not included these preliminary results in the manuscript but can add them if the editor feels it is appropriate.

-The authors did not demonstrate whether bNACHT25 is soluble or insoluble in the absence of DnaJ.

We thank the reviewer for this suggestion. We have now provided data demonstrating that bNACHT25 is soluble in the presence and absence of DnaJ (S7A Fig.)

The use of the hyperactive stimulus-independent mutant does not prove that the folding of bNACHT25 WT is independent of DnaJ/K/E. In addition, it is not clear whether the WT and the hyperactive mutant are comparably expressed.

We have now provided data demonstrating that bNACHT25 and bNACHT25^H506L^ are comparably expressed in the presence and absence of *dnaJ* (S7B Fig.)

-The fact that the DnaK/J/E chaperone machine is involved in this process, and not only DnaJ, is interesting but as for DnaJ, the mechanism of sensing is not provided (i.e., does it mediate bNACHT folding, sequestration or degradation in the absence of phage, do phage proteins compete with bNACHT for binding to DnaJ, etc..).

We acknowledge that the full sensing mechanism has yet to be discovered, and for this reason, have changed our submission to a “Discovery Report.” This is a format designed for interesting studies that may not have a complete molecular mechanism. We have also now included a paragraph within the discussion to explain the limitations of our study to acknowledge alternative hypotheses and outstanding questions like those described (Lines 384-393). Finally, while our IPs suggest that bNACHT25 and DnaJ are found in the same complex, it is true that their interaction may not be direct. We have adjusted our model (Fig. 5F) to represent this uncertainty.

Reviewer #4

In this revised manuscript, the authors have added some experiments and addressed some of the reviewers' comments. However, they have not solved the fundamental problem with the paper. The novelty that the authors are trying to push here is that bNACHT25/32 is "guarding a host cell process rather than binding a specific phage-derived molecule." The authors have still provided no direct proof for this idea. Even the indirect proof is weak.

We disagree that the proof is weak but acknowledge that the full mechanism has yet to be fully elucidated. For this reason, have changed our submission to a “Discovery Report.” This is a format designed for interesting studies that may not have a complete molecular mechanism. We have also now included a paragraph within the discussion to explain the limitations of our study to acknowledge alternative hypotheses and outstanding questions like those described (Lines 384-393).

In their abstract, the authors claim:
1. "Here we determine the mechanism of RNA phage detection by the bacterial NLR-related protein bNACHT25 in E. coli." This is not true. The escaper mutants suggest a role for CP. However, the authors could not demonstrate a direct interaction between bNACHT and CP. CP and bNACHT co-expression from plasmids induced DNA degradation and cell death. However, the plasmid assay is non-physiological because both proteins are produced at much higher levels than in the experiments testing phage replication. The authors have not shown that the bNACHT system kills cells during phage infection, or that the DNA degradation observed in the plasmid assay is the mechanism for blocking phage replication. They have not shown that the detection of DNA degradation activity (i.e. 40 min after infection for bNACHT32) correlates with the MS2 infection cycle (i.e. how long after phage infection are infectious phage particles produced). So, we do not know how bNACHT is detecting phage infection and we do not know how it is blocking phage infection.

We have shown (1) DNase activity of bNACHT25/32 is activated by phage infection (Fig. 1E), (2) DNase activity is required for phage defense (Fig. 1B), and (3) stimulus-independent activation of bNACHT25 causes DNA degradation (Fig. 5A-B) and decreases the recovered CFU (Kibby, et al., 2023). We do not claim that bNACHT25 or bNACHT32 induce cell death as cell death is a complicated and polarizing topic. Rather, we demonstrate that DNase activation is growth inhibiting. Our data relies on mutations disrupting the catalytic activity of bNACHT proteins, and where possible, bNACHT levels are kept consistent between phage and co-expression assays.

Nevertheless, we have altered the language we use in our abstract to try and better reflect our data (Lines 28-29).

Supplementary Figure 2 clearly shows the timeline for lysis after phage infection and can be compared with data in the remainder of the manuscript. We know that bNACHT25 is blocking phage infection through its DNase activity for reasons highlighted above. The focus of our manuscript is on uncovering the phage-sensing mechanism.

2. "A genetic assay confirmed CP was sufficient to activate bNACHT25 but the two proteins did not directly interact." CP is not proven to be sufficient for activation during phage infection. On the other hand, the non-detection of a CP:bNACHT interaction using co-IP is not conclusive proof of anything. A strong transient interaction with CP (i.e. fast on- and off-rates) or one that is too weak to detect by co-IP may be sufficient to activate bNACHT.

We have altered this sentence accordingly (Line 26).

We have now included a section in the discussion to describe the limitations of our study (Lines 384-393) to discuss potential alternative hypotheses.

3. "Instead, we found bNACHT25 requires the host chaperone DnaJ to detect CP." The authors have shown that DnaJ is required for functioning of the bNACHT system. They have not in any way shown that DnaJ is required to detect CP. For this conclusion, the authors are relying on a new result: "We now provide a crucial experiment to demonstrate that the bNACHT25 protein is fully functional in dnaJ-deficient cells (Fig 5D and 5E). We have used an inducible system to express a hyperactive allele of bNACHT25. In these experiments, bNACHT25 is active in a stimulus-independent manner and disruption of dnaJ did not impact nuclease activity (Fig 5D and 5E). These data complement data already found in the manuscript showing that bNACHT25 is similarly expressed in wild-type and dnaJ-mutant strains (Fig 4C)." This experiment is not convincing. First, there is actually less DNA degradation by the H506L mutants in the dnaJ-deficient strain, so it is not fully functional. Second, this assay is non-physiological, and measures only the DNA degradation activity of NACHT25. It does not determine whether it can still bind to another protein and be activated.

We have also now shown that levels of soluble bNACHT25 and stimulus-independent bNACHT25 are equivalent in the presence or absence of *dnaJ* (S7A Fig). We hope that the reviewer will find we have shown in *some* way that DnaJ is required.

See response to Reviewer #3, Comment 1, for a detailed description of our efforts to construct an *in vitro* assay.

4. "Our data suggest that bNACHT25 detects a wide range of phages by guarding a host cell process rather than binding a specific phage-derived molecule." There is no evidence at all that NACHT25 guards a host cell process involving DnaJ. A fundamental problem with this statement is that the authors provide no data to show that MS2 infection has any impact on DnaJ. In my previous review, I suggested that MS2 infection may increase DnaJ expression. However, the authors provided new data to show that this is not true. In the discussion, the authors push their idea further saying, "bNACHT25 adopts a mechanism of sensing most similar to the guard model and senses perturbations to the host cell proteome. CP is not a conventional pathogen effector; however, it still impacts a core host process that bNACHT25 monitors as a proxy for infection." This speculation goes way beyond any data presented here since the authors have not identified any change in a DnaJ-related process that might be detected. They have not even shown that CP binds to DnaJ, which might support this idea.

We have adjusted our language to highlight that we have not elucidated the full sensing mechanism of bNACHT25 (Lines 384-393). Accordingly, we propose a potential guard model in the discussion section, as the purpose of the discussion section is to speculate on potential models and implications of our data. We believe that our data fits with this model, despite not knowing the full molecular details. Importantly, we also show that MS2 requires *dnaJ* for replication, which demonstrates an unambiguous connection even if we do not fully know why.

The most important finding in this paper, as described in the title, is that DnaJ mediates the activity of these bNACHT proteins. Given that DnaJ could be affecting bNACHT activity through many mechanisms (most likely by stabilizing or helping to form its active conformation), the onus is on the authors to provide strong evidence for their conclusion, which would be very exciting if true. However, the only evidence they have for this conclusion is their inability to detect a direct interaction between NACHT and CP, and their flawed experiment showing that bNACHT is still active in the absence of DnaJ. This is not enough.

There appears to be a misunderstanding over the hyperactive bNACHT25 and DnaJ. If upstream activation of bNACHT25 by phage and phage proteins is completely blocked when *dnaJ* is knocked out, but downstream, stimulus-independent activation by the H506L mutation still allows DNA degradation, this supports our model that DnaJ is functioning in sensing. We have also now shown that bNACHT25^H506L^ and bNACHT25^WT^ are comparably expressed (S7B Fig).

Thus, I feel strongly that publishing this manuscript in its current form and with its current emphasis would be very misleading. I am not saying that the authors' hypothesis is wrong, but publishing it will require insight into the mechanism. The pathway way to this insight will likely require a greater understanding of how bNACHT and DnaJ affect the MS2
lifecycle (i.e. do more phage infection experiments). Given that this may entail considerably more experiments, the authors might consider publishing the current data, but with a different emphasis.

We hope the reviewer finds that the changes to language in the revised manuscript sufficiently explain the limitations of our study.
